# The regulatory role of *ZmSTOMAGEN1/2* in maize stomatal development is elucidated via gene editing and metabolic profiling

**DOI:** 10.1371/journal.pone.0328433

**Published:** 2025-07-14

**Authors:** Juan-Juan Xu, Qing-Yun Chen, Li-Fang Qin, Yuan Liu, You-Zhi Li, Xian-Wei Fan

**Affiliations:** State Key Laboratory for Conservation and Utilization of Subtropical Agro-bioresources, College of Life Science and Technology, Guangxi University, Nanning, Guangxi, China; Institute of Agricultural Resources and Regional Planning, Chinese Academy of Agricultural Sciences, CHINA

## Abstract

Stomatal development is mediated by EPIDERMAL PATTERNING FACTORs (EPFs), a family of secreted peptides including STOMAGEN/EPFL9 in *Arabidopsis*. To clarify the functional role of STOMAGEN orthologues in maize (*Zea mays*), we generated a double knockout mutant of ZmSTOMAGEN1 and ZmSTOMAGEN2 using CRISPR/Cas9 system. Comprehensive phenotypic analysis revealed that the *zmstomagen1/2* mutant exhibited severe stomatal development defects, including complete absence of stomata between epidermal cells in stomatal lineage files and abnormal stomatal complexes with small lobed cells. These aberrant cells likely arose from failed asymmetric divisions of guard mother cells, ultimately preventing the formation of functional stomatal complexes. A double knockout of *ZmSTOMAGEN1/2* reduced the expression of *SPEECHLESS1* (*SPCH1*), *MUTE*, *SCREAM2* (*SCRM2*), and STOMATAL DENSITY AND DISTRIBUTION1 (*SDD1*), impairing stomatal initiation and cell fate transition in early stomatal lineage cells. The mutant displayed a lower stomatal density and index, leading to reduced net photosynthetic rate, transpiration rate, and stomatal conductance but increased water-use efficiency (WUE). Compared to the wild-type plants (HiII-A × HiII-B), the *zmstomagen1/2* mutant exhibited significant alterations in phytohormone homeostasis. These included brassinosteroid metabolite imbalance (increased typhasterol, decreased castasterone) and differential gibberellin regulation (elevated GA4, reduced GA1). These hormonal perturbations suggest that impaired stomatal morphogenesis in *zmstomagen1/2* mutants result from disrupted crosstalk between multiple hormonals signaling networks. Our findings reveal a crucial role for ZmSTOMAGEN1/2 in regulating cell fate decisions within the stomatal lineage and provide a potential strategy for enhancing WUE in maize by manipulating *ZmSTOMAGEN1/2* expression.

## Introduction

Stomata are composed of guard cells and small pores that regulate gaseous exchange between plants and the atmosphere. They are essential for photosynthesis in most plants and play a pivotal role in global carbon cycling and water cycling [1]. The morphology, structure, and distribution of stomata vary substantially between *Arabidopsis* and grasses. The stomatal complex of grasses consists of two guard cells that are narrow, elongate, and have rather thickened walls, flanked by two subsidiary cells, which are distinct from other epidermal cells and stomata of dicotyledonous plants. Meanwhile, cultivated monocotyledonous grass varieties significantly contribute to human nutrition [2]. Therefore, further research on grass stomata holds promise for enhancing crop production and supporting food security.

Over the past two decades, our knowledge of stomatal development has mainly been derived from *Arabidopsis* plants. In *Arabidopsis*, stomatal formation initiates with an asymmetric division of a protodermal cell, generating a meristemoid mother cell (MMC) and a stomatal lineage ground cell (SLGC). The MMC subsequently undergoes asymmetric divisions, ultimately differentiating into guard cells that form the mature stomatal complex. In contrast to the dicot *Arabidopsis*, stomatal development in grasses exhibits a distinct pathway. The stomatal lineage in grasses is initiated by an asymmetric entry division of a protodermal cell, producing a guard mother cell (GMC). The GMC then recruits neighboring subsidiary mother cells (SMCs), which undergo asymmetric divisions to form subsidiary cells flanking the GMC. Finally, the GMC divides symmetrically to produce two guard cells, completing the stomatal complex. Three paralogous basic helix-loop-helix (bHLH) transcription factors, SPEECHLESS (SPCH), MUTE, and FAMA, regulate key stages of stomatal lineage progression, controlling entry, proliferation, and terminal differentiation, respectively. These factors interact with the functionally redundant INDUCER OF CBF EXPRESSION1 (ICE1) and SCRM2 [3–5].

While the function of *FAMA* in cell fate specification and stomatal development appears to be conserved in rice and maize, the roles of *MUTE* and the two *SPCH* paralogs have diverged [6]. In *Arabidopsis*, *AtSPCH* drives asymmetric divisions in the stomatal lineage. In contrast, *Brachypodium distachyon* employs a modified regulatory network where duplicated *SPCH* paralogs (*BdSPCH1/BdSPCH2*) work with distinct ICE/SCRM (INDUCER OF CBF EXPRESSION/SCREAM) partners. While *BdICE1* controls initial asymmetric divisions, *BdSCRM2* governs later differentiation events [7]. This divergence in transcriptional regulation highlights alternative evolutionary strategies for stomatal patterning in monocots versus dicots. The suppressor of cAMP receptor (SCAR)/WASP family verprolin homologous (WAVE) complex polarized Pangloss1 (PAN1) receptors to induce the asymmetric division of subsidiary mother cell in maize [8]. Despite extensive descriptions of transcriptional regulatory signals in dicotyledons, the regulation of stomatal development in grasses remains poorly understood.

Extracellular epidermal patterning factors (EPFs) are key signaling peptides that regulate the precise spacing of stomatal precursor cells by binding to ERECTA-family receptors in *Arabidopsis* [9–11]. *EPF1* and *EPF2* act as negative regulators of stomatal formation, reducing stomatal number in *EPF1*/*EPF2*-overexpressed *Arabidopsis* [9]. Similarly, overexpression of *HvEPF1* limits stomatal development and improves water use efficiency in grasses [12], while *OsEPF1/2* overexpression decreases stomatal density and conductance in rice [13]. In contrast to EPF1/2, STOMAGEN/EPFL9, derived from leaf mesophyll tissues, functions as a positive regulator of stomatal differentiation [14,15]. Overexpression of *PagSTOMAGEN* positively regulates stomatal density and increases the photosynthetic rate in poplar [16], while heterologous expression of *FSTOMAGEN* (or *AFSTO*) in *Arabidopsis* also boosts stomatal density [17]. Conversely, the knockout of *OsSTOMAGEN* and *OsEPFL10* maintains 25% and 80% of stomatal density of wild-type rice, respectively [18]. Loss of *OsEPFL9−1* also results in an eight**-**fold reduction in stomatal density in rice [19]. Additionally, *BdSTOMAGEN* can promote stomatal development at various stages in grass [20]. These studies highlight the potential role of manipulating EPF/STOMAGEN levels to optimize stomatal density and improve drought tolerance in grasses.

In this study, we utilized the CRISPR/Cas9 gene-editing system to explore the role of maize *STOMAGEN1/2* in stomatal development. We also examined how reduced stomatal density in the *zmstomagen1/2* double mutant impacts stomatal conductance, carbon assimilation, and water conservation. Our findings suggest that *ZmSTOMAGEN1/2* positively regulates stomatal number and underscore the potential of gene-editing strategies for improved water-use efficiency.

## Materials and methods

### Plant growth conditions

Wild-type (HiII-A × HiII-B) and T3 transgenic maize seeds were sown in pots (10 cm length × 8 cm width × 12 cm depth) containing a 1:1:1 (v/v) mixture of soil, peat, and vermiculite. Plants were grown in the greenhouse under controlled conditions with a 14-h photoperiod, day/night temperature of 28°C/15°C relative humidity of 60 ± 5%, and photosynthetic photo flux density (PPFD) of 150 μmol m^−2^ s^−1^. Leaf samples were collected from five-leaf stage seedlings for RNA extraction, microscopy, and metabolite profiling.

### Bioinformatics analysis of the *ZmSTOMAGEN* gene

The amino acid sequences of ZmSTOMAGEN and its homologous were compared using DNAMAN. A molecular phylogenetic tree was constructed based on the neighbor-joining method in MEGA version 7.0.

### RNA extraction and cDNA synthesis

Total RNA was extracted using the RNA iso Plus (TaKaRa, China) and homogenized with a hand drill and micropestle on dry ice. RNA was then isolated and precipitated according to the TaKaRa Reagent product protocol. After RNA extraction, gDNA Eraser (TaKaRa) was used to degrade contaminating genomic DNA following the manufacturer’s recommendations. Complementary DNA (cDNA) was synthesized from total RNA using the Prime Script RT Enzyme Mix I System (TaKaRa) and RT Primer Mix.

### Construction of the sgRNA-Cas9 expression vector

To target the *ZmSTOMAGEN1* (GRMZM2G170975) and *ZmSTOMAGEN2* (GRMZM2G115133) marker sites in maize, we generated plasmids pYLCRISPR/Cas9Pubi-B-ZmSTOM4T according to previous methods [21], with slight modification. In summary, the promoter of OsU6a was amplified from the plasmid pYLsgRNA-OsU# template, using two primers (U-F and T1-R). The sT1gRNA scaffold was amplified with another two primers (T1-F and gR-R). Two PCR fragments were then used as templates to perform overlapping PCR to generate OsU6a-sT1gRNA using primers (Pps-GGL and Pgs-GG2). Other three similar PCR products, OsU6b-sT2gRNA, OsU6c-sT3gRNA, and OsU3-sT4gRNA, were generated as described above, and all the primers are listed in Table 1. Total PCR products were purified and diluted to a concentration of 20 ng µL^-1^. For cloning, restriction-ligation reactions (15 µL) were set up using 1.5 µL of NEB 10 × ligation buffer, 10 U of BsaI, 35 U of T4 DNA ligase (Takara), 60–80 ng of the intact binary plasmid pYLCRISPR/Cas9Pubi-B and the purified products (20 ng for each sgRNA expression cassette) supplemented with ddH_2_O to a volume of 15 µL. The reactions were incubated for 3 cycles (37 °C, 10 min; 10 °C, 5 min; 20 °C, 5 min) and 10 cycles (37 °C, 3 min; 10 °C, 5 min; 20 °C, 5 min) and then maintained at 37 °C for 5 min. The reaction products were transformed into *Escherichia coli* DH5α and the positive strains selected for identification using two primers (SP-L1/SP-R).

**Table 1:** Primers used for construction of the sgRNA-Cas9 expression vector.

Primers	Sequence (5’ to 3’)
T1-F	gccGCTCACTTGAACAAGAGGAA
T1-R	aaacTTCCTCTTGTTCAAGTGAG
T2-F	gttgCAGGGAGCTTGTGGTAGTCG
T2-R	aaacCGACTACCACAAGCTCCCTG
T3-F	tcagTACTGCTGAGCAAGTCCCAG
T3-R	aaacCTGGGACTTGCTCAGCAGTA
T4-F	ggcAGTGGTAAGCACTGTTCATG
T4-R	aaacCATGAACAGTGCTTACCAC
U-F	CTCCGTTTTACCTGTGGAATCG
gR-R	CGGAGGAAAATTCCATCCAC
SP-L1	GCGGTGTCATCTATGTTACTAG
SP-R	CCCGACATAGATGCAATAACTTC
Pps-GGL	TTCAGAggtctcT**ctcg**ACTAGTATGGAATCGGCAGCAAAGG
Pgs-GG2	AGCGTGggtctcG**tcag**ggTCCATCCACTCCAAGCTC
Pps-GG2	TTCAGAggtctcT**ctga**cacTGGAATCGGCAGCAAAGG
Pgs-GG3	AGCGTGggtctcG**tctt**cacTCCATCCACTCCAAGCTC
Pps-GG3	TTCAGAggtctcT**aaga**cttTGGAATCGGCAGCAAAGG
Pgs-GG4	AGCGTGggtctcG**agtc**cttTCCATCCACTCCAAGCTC
Pps-GG4	TTCAGAggtctcT**gact**acaTGGAATCGGCAGCAAAGG
Pgs-GGR	AGCGTGggtctcG**accg**ACGCGTATCCATCCACTCCAAGCTC

### Agrobacterium-mediated maize transformation

Maize HiII-A and HiII-B seeds were planted in an experimental field in Nanning from March to July. The F1 immature zygotic embryos were harvested at 11 days after pollination (HiII-A × HiII-B) and used for Agrobacterium-mediated transformation following methods reported by Vega et al., (2008) [22], with slight modification. In brief, immature ears were stored at 4 °C for 3 days before embryo dissection, and then the immature embryos were infected with Agrobacterium for 10 min, and then placed on co-cultivation medium at 20 °C in the dark for 3 days. Subsequently, embryos were transferred to resting medium at 28 °C in the dark for 7 days and transferred to selection medium D1 for 2 weeks under conditions similar to the resting medium. In addition, the growing embryogenic calluses were sub-cultured on the selection medium D2 for 2 months. The resistant transformed calluses were divided into small pieces and maintained in the dark at 28 °C for 2 weeks. Maturation took place when opaque calluses occurred after culturing on the medium E for 2–3 weeks at 25 °C (dark), and then embryogenic calluses were harvested for regeneration on the medium F under a 16-h/8-h light/dark cycle at 25 °C. Finally, the transgenic plants (T0) were transplanted into a greenhouse with the temperature maintained at 28 °C. Since tasseling and silking did not occur simultaneously, pollen from the wild-type maize (HiII-A × HiII-B) was used to perform pollination in the T0 ears (in-crossing) to generate hemizygous seeds (T1). The T1 seeds were planted and pollinated (selfing) to produce T2 seeds, and T2 seeds were planted and pollinated (selfing) to produce T3 seeds. T3 seedlings were used to carry out the stomatal development studies.

### DNA extraction and PCR verification

Genomic DNA was isolated from leaves of BAR-positive transgenic lines using Plant Genomic DNA Kit (CWBio, China) according to the manufacturer’s instructions. The transgenic analysis was performed by PCR with specific primers Cas9‐F 5′-GAAGCTCAAGTCCGTCAAGG-3′, Cas9‐R 5′-GCAAGCTCGTTACCCTTCTG-3′. The PCR amplicons were analyzed by electrophoresis on a 1% (w/v) agarose gel. Positive transgenic lines should be visible as a bright band of approximately 250 bp.

### Mutagenesis analysis at target sites

Leaves of T3 transgenic lines were used for genomic DNA isolation. PCR amplification was carried out using primer pairs flanking the designed target sites. The genomic regions flanking *ZmSTOMAGEN1* target sites were amplified with the following primers: MuSTO1-F 5′-CCTGTTATCCCGTGCGTTC-3′, MuSTO1-R 5′-ACAAAATGCTCCTCTAGGCTC-3′. *ZmSTOMAGEN2* target regions were amplified with MuSTO2-F 5′-GTCTTCCTTTCCTGCTGTGC-3′and MuSTO2-R 5′-CGCCATAGGTCAACCGTACT-3′ primers. The PCR products were analyzed by electrophoresis on a 1% (w/v) agarose gel. Mutations in T3 plants were further identified by Sanger sequencing of the PCR products, and sequenced directly using internal specific primers whose binding positions were about 150–250 bp upstream of the target sites. *ZmSTOMAGEN1* PCR products were sequenced using the MuSTO1-Seq 5′-CATGGTGGTCTCCGCAGTC-3′ primer, while the *ZmSTOMAGEN2* sequencing primer was MuSTO2-Seq 5′-AGCCATTGCACCTGTCTCCC-3′.

### Quantitative PCR and data analysis

The base section (0–1 cm) of the 5th leaf was collected for RNA extraction when the 5th leaf was emerging from the whorl. Quantitative PCR was conducted using SsoAdvancedTM Universal SYBR®Green Supermix (Bio-Rad, Hercules, CA, USA) and cDNA templates (described above) on a CFX96 Real-Time PCR System (Bio-Rad Laboratories Inc., USA). The nonspecific false-positive amplifications were eliminated based on the dissociation curves of SYBR Green Supermix. Data were normalized to *ACTIN1* mRNA expression levels (internal control), and fold changes are displayed relative to the control plant lines using the comparative threshold cycle method. Error bars represent standard deviations of technical replicates (n = 3). Gene-specific RT-qPCR primers are listed in S1 Table.

### In-situ hybridization

In-situ hybridization was performed as described previously [23], with a few modifications. The one-leafed young seedlings were collected and DIG-labelled probes were prepared from the coding region of the *STOMAGEN* cDNA (306 bp). Antisense and sense probes used in the present study were generated via PCR with T7 and T3 promoter adding primers (S1 Table). RNA in situ hybridization procedures was carried out following the RNA hybridization protocol. Slides were observed and photographed using a Zeiss Axio Scan microscope (Carl Zeiss Jena, Germany).

### Observation of stomata

The abaxial surfaces of sectored leaves were collected at the zones of maximum leaf width, near the central veins, and then fixed in 95% ethanol:glacial acetic acid (3:1, v: v) for 30 min. The samples were subsequently stained with Toluidine Blue O (0.03% in water) according to the method described by [24]. The stomata of the abaxial epidermis obtained were examined using a light microscope (ECLIPSE E100, Nikon) equipped with a digital camera (SBI Investment Co., Ltd.). Three microscopic fields were randomly selected for analysis per replicate.

Stomatal density was calculated as the number of stomata per unit area. The stomatal index (%) was determined using the formula:


$$\begin{array}{c} Stomatalindex(\%)=\frac{Stomatanumber}{Stomatanumber+pavementcellnumber}\backslash \times 100\% \end{array}$$

### Propidium iodide staining

Basal leaf segments (1 cm in length) were cut into 0.2 cm wide × 0.5 cm long strips and fixed in a 4% paraformaldehyde solution for 20 min at room temperature. The plant tissues were rinsed four times with PBS for 10 min each time. Next, 200 µL of the prepared PI working solution (Yeasen, Shanghai, China) was applied to cover the plant cells on the coverslips, and the cells were incubated at room temperature for 30 min. After incubation, the coverslips were washed three times with PBS for 5 min each. Images were acquired using a Leica SP8 Confocal Microscope.

### Gas exchange measurements

Net photosynthetic rate (Pn), transpiration rate (Tr), and stomatal conductance (gs) in the 5th uppermost fully expanded leaf in transgenic lines and wild type (HiII-A × HiII-B) were measured using a Li6400XT Portable Photosynthetic System (Li-Cor, Lincoln, NE, USA) when seedlings were in the jointing stage. Measurements were obtained between 0900 and 1100 h on clear sunny days when the photosynthetic photon flux density (PPFD) over the plant canopies was 1200 μmol m^−2^ s ^−1^. PPFD was measured with a 6400-02B LED Red/Blue Light Source (Li-Cor, Lincoln, NE, USA). Instantaneous water use efficiency (iWUE) was calculated as the ratio of Pn to gs.

Light curves were measured using the AutoProgram function according to Lobo et al., (2013), with slight modification. Light intensity was set at 2,000, 1,600, 1,200, 1,000, 800, 600, 400, 300, 200, 150, 100, 50 and 0 μmol (photon) m^–2^ s^–1^ and supplied by an artificial light source, Li-6400-02B (LI-COR Inc., Lincoln, USA), with a minimum wait time of 120 s and a maximum wait time of 200 s. Photosynthetic PAR (Pn-PAR) curves were fitted using a non-rectangular hyperbola model [25] according to the recorded data in different maize lines. Maximum net photosynthetic rate, dark respiration rate, light compensation point, and light saturation point were estimated based on the trends of the measured curve, and the apparent quantum yield was obtained using the linear regression method of the Pn-PAR curve under weak light conditions [PAR ≤ 200 μmol (photon) m^–2^ s^–1^].

### Metabolite extraction and analysis

The base (0−1 cm) of the 5th leaf from the base of 3-week-old maize plants (at which point the 5th leaf is emerging but not yet fully expanded) were harvested and immediately ground into a fine powder using a pre-cooled mortar and pestle in liquid nitrogen. Approximately 250 mg of the powdered tissue was weighed and transferred into pre-chilled tubes. Metabolites were extracted by adding 1 mL of cold 75:20:5 (v/v/v) methanol: ddH_2_O: formic acid solution (30 μg mL^-1^ DDTC) to each tube. The samples were vortexed for 1 min and then incubated at 4 °C for 12 h. Following incubation, the samples were centrifuged at 10000 × g for 15 min at 4 °C. The supernatant was carefully collected and transferred to clean tubes. The collected supernatant was concentrated and dried in a vacuum concentrator (Eppendorf concentrator 5301, Eppendorf AG 22331, Hamburg, Germany) at 45 °C. The dried extract was reconstituted in 400 µL of methanol and then filtered through 0.22 µm syringe filters. The filtered extracts were analyzed using a liquid chromatography-quadrupole Exactive Orbitrap tandem mass spectrometry system (UHPLC-Q Exactive Orbitrap MS/MS; Thermo Fisher Scientific) equipped with a heated electrospray ionization (HESI) source. The system was operated in both positive and negative ionization modes. Chromatographic separation was achieved using a binary mobile phase system of water containing 0.1% formic acid (solvent A) and methanol (solvent B). The gradient elution program was: 0−2 min, 95% A; 2−13 min, 95–0% A; 13−16 min, 0% A; 16-16.1 min, 0−95% A; and 16.1−19 min, 95% A. The injection volume was 2 μL with a flow rate of 0.3 mL min^-1^. The column compartment and autosampler tray were maintained at 30°C and 10°C, respectively.

Mass spectrometry parameters were optimized as follows: spray voltage, 3 kV; capillary temperature, 320°C; sheath gas flow rate, 35 arbitrary units; and auxiliary gas flow rate, 10 arbitrary units. Nitrogen served as both sheath and auxiliary gas, with the auxiliary gas heated to 350°C to enhance solvent evaporation and ionization efficiency. Data were acquired in Full MS and Full MS/data-dependent MS2 (Full MS/DD–MS2) scanning modes across a mass range of 20–2000 m/z, with resolution settings of 70,000 and 17,500 for primary and secondary scans, respectively.

For data processing and metabolite identification, raw data files were analyzed using Compound Discoverer 3.2 software (Thermo Fisher Scientific). Metabolite abundances were normalized via Z-score transformation and visualized through heatmap to compare metabolite profiles between WT (HiII-A × HiII-B) and *zmstomagen1/2* mutant plants. Differential metabolites were identified using stringent criteria (|log₂ fold change| ≥ 1 and adjusted *p* ≤ 0.05), then plotted as volcano plots. Upregulated metabolites highlighted in red and downregulated in blue. Pathway enrichment analysis was performed using hypergeometric tests (*p* ≤ 0.05) against the Kyoto Encyclopedia of Genes and Genomes (KEGG) database, with results visualized as bar plots indicating enrichment significance (color gradient) and pathway impact scores (bar height).

### Statistical analysis

All the data were presented as averaged values of three independent replicates. Statistical analyses of the data were carried out using a student’s t-tests, and multiple comparisons of means were analyzed using Tukey’s test. Differences were considered statistically significant at *P* < 0.05. These analyses were performed using SPSS 13 (SPSS Inc., Chicago, IL, US) and GraphPad Prism version 9.0.

## Results

### CRISPR/Cas9 system construction of homologues of STOMAGEN and transformation into maize

**Fig 1:**
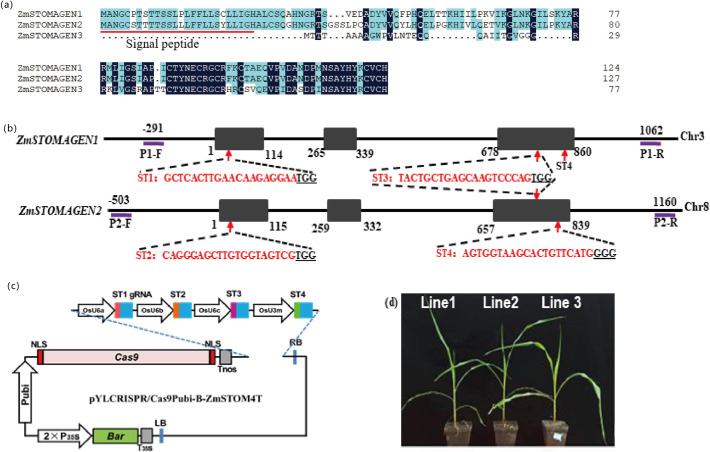
Schematic diagram of CRISPR/Cas9 system construction and transformation. (a) A comparative analysis of the protein sequence of ZmSTOMAGEN1, ZmSTOMAGEN2 and ZmSTOMAGEN3. ZmSTOMAGEN1 and ZmSTOMAGEN2 share 86.99% sequence similarity, especially in the conserved functional domain (amino acid positions 79-123 of ZmSTOMAGEN1). However, these three ZmSTOMAGEN homologs are located on distinct chromosomes. (b) Schematic illustration of target genes and relative positions of gRNA binding sites in *ZmSTOMAGEN1* and *ZmSTOMAGEN2*, respectively. Black box represents exons of *ZmSTOMAGEN1* gene and *ZmSTOMAGEN2* gene, respectively. Red arrow presents target site and the red sequence means the target sequence. Underline shows the PAM site for the gRNAs. Two pairs of primers (P1-F/P1-R) was designed to identify the sequence of *ZmSTOMAGNEN1*; P2-F/P2-R was used to identify the sequence of *ZmSTOMAGEN2*. The number represents nucleotide position. (c) Illustration of cloning of four sgRNA expression cassettes into CRISPR/Cas9 binary vectors by single Golden Gate ligation. (d) Transgenic plants (T0) were generated through the progeny of Hi-II maize immature embryos mediated by *Agrobacterium tumefaciens.*

To explore the function of STOMAGEN in grasses, we identified three homologous proteins in maize: ZmSTOMAGEN1 (GRMZM2G170975/Zm00001d012079), ZmSTOMAGEN2 (GRMZM2G115133/Zm00001d042381), and ZmSTOMAGEN3 (GRMZM2G017321/Zm00001d049795). Among these, ZmSTOMAGEN2 (GRMZM2G115133) was not previously described by Zhao et al. (2022) [17], suggesting it represents a novel maize STOMAGEN ortholog identified in our current study. Protein sequences were retrieved from the Ensembl Plants database (http://plants.ensembl.org/), and a phylogenetic tree was constructed. The results showed that ZmSTOMAGEN1 and ZmSTOMAGEN2 shared high similarity, especially in their conserved domains (Figs 1a and S1). *ZmSTOMAGEN1* and *ZmSTOMAGEN2* are located on the 8^th^ and 3^rd^ chromosomes, respectively. To target these genes, we designed three guide RNA (gRNA) sites in the first and third exon of *ZmSTOMAGEN1* and *ZmSTOMAGEN2* (Fig 1b) using the CRISPR-GE tool (http://skl.scau.edu.cn/). The SgRNAs (sT1gRNA, sT2gRNA, sT3gRNA, and sT4gRNA) were driven using rice U6 and U3 promoters, and Cas9 was driven using the maize ubiquitin promoter (Pubi) (Fig 1c) [21]. The sgRNA-Cas9 expression plasmid was transformed into maize immature embryo to generate the transgenic plants according to the method described by Vega *et al.*, (2008) [22]. From 1,000 immature maize embryos, 200 resistant embryogenic calluses were selected using a medium with 3 mg L^-1^ bialaphos, and then 39 putative transgenic lines were identified and moved to the greenhouse for further evaluation (Fig 1d). To verify the specificity of our CRISPR/Cas9 editing, we examined the expression of maize *EPFL4* orthologs, which exhibit the highest sequence similarity to *ZmSTOMAGEN1/2* among the EPF/EPFL family members (S1 Fig) and therefore represent the most likely candidates for potential off-target effects of our sgRNAs.

**Fig 2:**
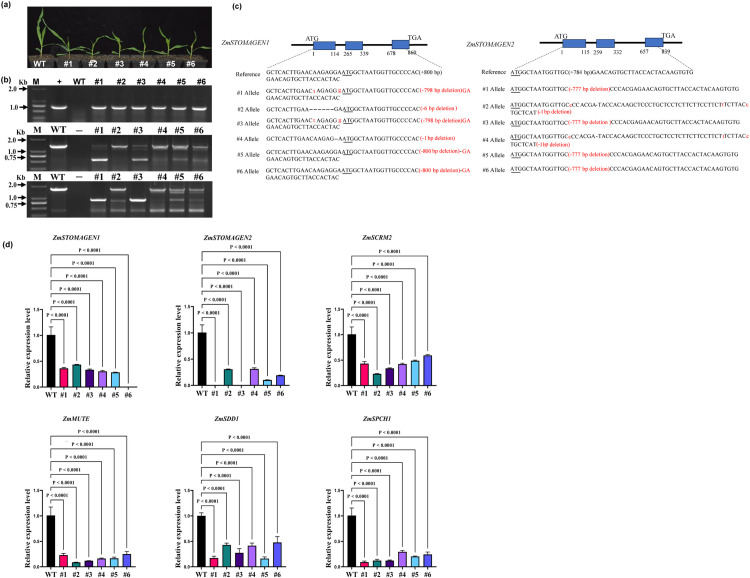
CRISPR/Cas9 –induced mutations in *ZmSTOAMAGEN1* and *ZmSTOMAGEN2* in T3 mutants. (a) Seedlings of wild type and mutant maize at 12 days post-sowing. (b) Specific and non-specific products of Cas9, *ZmSTOMAGEN1* and *ZmSTOMAGEN2* were identified by specific PCR amplification in mutants. The “+” symbol represents the amplification product (841 bp product) obtained from the universal primer PCR using the empty vector pYLCRISPR/Cas9Pubi-B. The *ZmSTOMAGEN1* and *ZmSTOMAGEN2* gene fragments in wild-type (WT) plants were amplified using the corresponding primer pairs P1-F/P1-R and P2-F/P2-R, resulting in 1353 bp and 1663 bp products, respectively. The “-” symbol denotes the negative control, which contained water instead of maize DNA. (c) Sequence analysis of CRISPR/Cas9-induced mutations in *ZmSTOMAGEN1* and *ZmSTOMAGEN2*. Upper panels: Gene structures of *ZmSTOMAGEN1* and *ZmSTOMAGEN2*. Middle panels: Reference wild-type sequences. Lower panels: Mutant sequences with insertions and deletions (highlighted in red) identified by Sanger sequencing. Red text indicates insertions and deletions relative to the wild-type sequence. (d) Transcripts of *ZmSTOMAGEN1/2* and pivotal regulators participating in stomatal development using the RT-qPCR assay in mutants. Data are expressed as means ± SD (n = 3). Results were analyzed using one-way ANOVA with Tukey’s post hoc tests at 95% confidence intervals.

The effects of editing *ZmSTOMAGEN1* and *ZmSTOMAGEN2* were evaluated by PCR amplification and sequencing of T3 transgenic plants (Fig 2). PCR analysis of genomic DNA from young leaves of T3 revealed mutated sequence band in agarose gel (Fig 2b and c). Purified PCR products were sequenced to identify the insertion or deletion event, confirming correct targeting of all alleles by alignment with wild-type templates. Most mutations were due to frameshift or deletion errors within the genes, significantly altering protein sequences of ZmSTOMAGEN1 and ZmSTOMAGEN2 (Fig 2c). Additionally, lines #2 and #4 contained small deletions in the 5’UTR of *ZmSTOMAGEN1*: a 6 bp deletion in line #2 and a 1 bp deletion in line #4. Furthermore, expression levels of *ZmSTOMAGEN1* and *ZmSTOMAGEN2* decreased similarly in T3 lines (Fig 2d). To investigate the correlation between *zmstomagen1/2* mutants and stomatal development, we detected the transcript abundance of four key genes in the young leaf base of *zmstomagen1/2* mutants (Fig 2d). Notably, the expression of three transcription factors (*ZmSPCH1*, *ZmMUTE*, and *ZmSCRM2*) was significantly down-regulated in the T3 *zmstomagen1/2* mutants (Fig 2d), as was the expression of *ZmSDD1* (Fig 2d).

### RNA in situ hybridization

**Fig 3:**
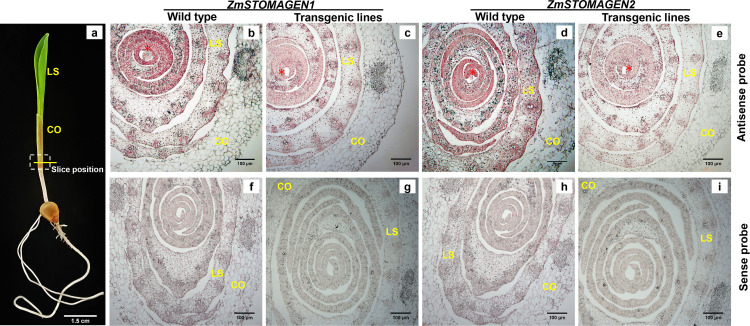
RNA expression patterns of ZmSTOMAGEN1/2 in wild-type maize and T3 transgenic seedling during early leaf development. (a) Wild-type maize seedlings 12 days after sowing. The yellow line represents the slice position in the dotted diagram. CO, coleoptile; LS, leaf sheath; wild-type (HiII-A × HiII-B). Cross section of leaf development from inside to outside around meristem apex in maize. The expression pattern of *ZmSTOMAGEN1* is gradually decreased with leaf development from inside (asterisk) to outside in wild type (b), and *ZmSTOMAGEN1* is more weakly expressed in transgenic line #1 (c) than in wild type plants. *ZmSTOMAGEN2* is strongly expressed in wild type (d) compared to in transgenic line #1 (e) Transcripts are undetectable in the sense control for *ZmSTOMAGEN1* and *ZmSTOMAGEN2* both in wild type (f, h) and transgenic line #1 (g, i). Asterisks indicate strong expression of *ZmSTOMAGEN1/2* was observed in the leaf around shoot apical meristem, but not in CO. Scale bars: 1.5 cm in a, 100 μm in b-i.

We further examined the expression patterns and localization of *ZmSTOMAGEN1/2* during the early stage of leaf development via RNA in situ hybridization, and then determined the expression level of *ZmSTOMAGEN1/2* gene in various tissue regions (T3, Fig 3a). In the wild type, *ZmSTOMAGEN1/2* transcripts were detected in the shoot apical meristem (SAM) and leaf primordia, with the strongest signals observed in the developing leaves surrounding the SAM. Expression of *ZmSTOMAGEN1/2* exhibited a consistent gradient, decreasing progressively from the SAM to the leaf sheath (LS) in both wild-type and transgenic plants (Fig 3b–e), contrasting with the sense probe controls (Fig 3f–i). This gradient of *ZmSTOMAGEN1/2* expression along the leaf developmental axis correlated with the progression of stomatal lineage specification and differentiation, which initiated in the basal leaf zone and proceeded towards the tip. The presence of *ZmSTOMAGEN1/2* transcripts in early leaf primordia supported their role in regulating the initial stages of stomatal development. However, the *ZmSTOMAGEN1* transcripts decreased considerably in the transgenic lines compared to those of wild type (Fig 3b and c), especially for *ZmSTOMAGEN2* (Fig 3d and e). *ZmSTOMAGEN1/2* expression was not observed in the maize coleoptile.

### *zmstomagen1/2* mutants exhibited impaired symmetric GMC division, and absence of SMCs

**Fig 4:**
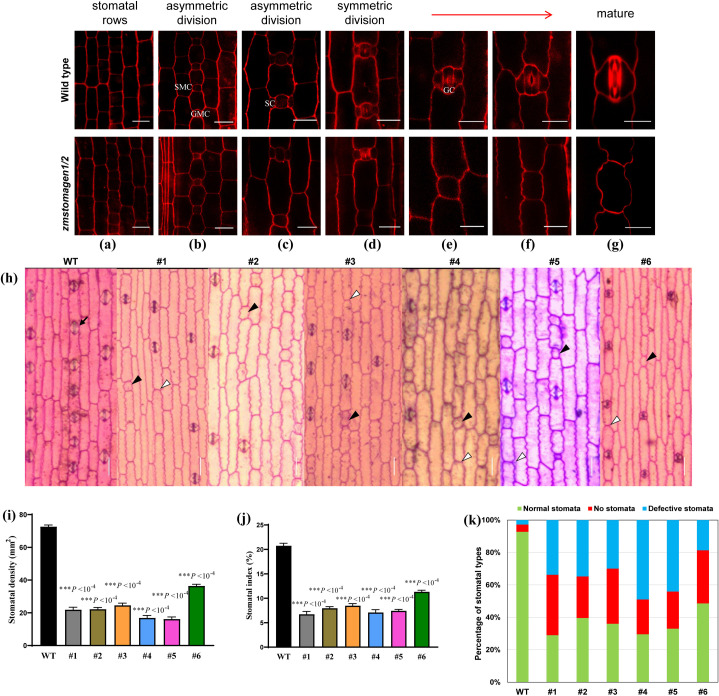
Variation in stomatal complex formation in maize leaves of wild type and T3 mutations. (a-g) Progression of stomatal development in wild-type (top) and zmstomagen1/2 mutant (bottom) plants, from stomatal rows to mature stomata. (a) stomatal rows, (b-c) asymmetric division, (d) symmetric division, and (g) mature stomata. Bar = 25 µm. (h) Toluidine blue O–stained epidermal peels from adult leaves of WT (HiII-A × HiII-B), and *zmstomagen1/2* mutants (#1, #2, #3, #4, #5, #6). Black arrowheads indicate normal stomatal complexes in the WT, while white and black triangles represent absent and defective stomatal complexes, respectively, in the mutant lines. Quantitative analysis of stomatal density (i), stomatal index (j) in mature leaf tissue. Error bars represent standard error of the mean (SE) (n > 1000 stomatal complexes were analyzed for each genotype). *P < 0.05, **P < 0.01, ***P < 0.001, ns represents no significance at *P* > 0.05, Independent-Samples T Test. (k) Quantitative analysis of abnormal stomatal complexes in mature leaf tissue (n > 1000 stomatal complexes were analyzed for each genotype). ‘No stomata’ indicates regions where stomatal files are present but mature stomatal complexes failed to develop, resulting in undifferentiated epidermal cells. ‘Defective stomata’ represents incomplete or malformed stomatal complexes.

Stomatal development in the wild-type maize leaf blade exhibited a dynamic, spatial gradient from the leaf base to the tip. In normal development, a terminal precursor (guard mother cell, GMC) was formed via initiating asymmetric division of protodermal cells (PDC) (Fig 4b). A neighboring cell received polarization signals from the newly formed GMC and underwent another asymmetric division, yielding a pair of stomatal precursor cells (SMCs) (Fig 4c). The SMCs differentiated into subsidiary cells (SCs), while the GMCs were divided to produce a pair of immature guard cells (GCs) through the symmetric division. Subsequently, the GCs matured to form a complete stomatal complex (Fig 4e). We further examined stomatal development in the *zmstomagen1/2* mutants. During the early stages of stomatal development, the *zmstomagen1/2* mutants exhibited normal processes of stomatal development, with PDCs successfully producing GMCs. In the *zmstomagen1/2* mutants, SMC formation and division were severely impaired, manifesting as disrupted formation and cytokinesis defects in SMCs. Abnormal GMCs accumulated markedly in mature mutant leaf tissues, accompanied by a defect in SMC recruitment and asymmetric division, ultimately preventing subsidiary cell differentiation (Fig 4c and d). In the *zmstomagen1/2* mutants (T3), multiple stomata were unable to develop between adjacent epidermal cells, and there was a significant reduction of stomatal numbers in mutants compared to the wild type (HiII-A × HiII-B). The absence of the more stomatal complexes between adjacent epidermal cells was observed in the mutants (Fig 4h, white arrowheads). Stomatal density was decreased by 63.1–76.7% (Fig 4i), while the stomatal index decreased by 47.9–71.4% (Fig 4j). The proportion of defective stomatal complexes was 7.15% in the wild type (HiII-A × HiII-B), but significantly increased to 64.02% in the *zmstomagen1/2* mutants (Fig 4k).

### Effect of stomatal variation caused by CRISPR/Cas9 system on gaseous exchange parameters

**Fig 5:**
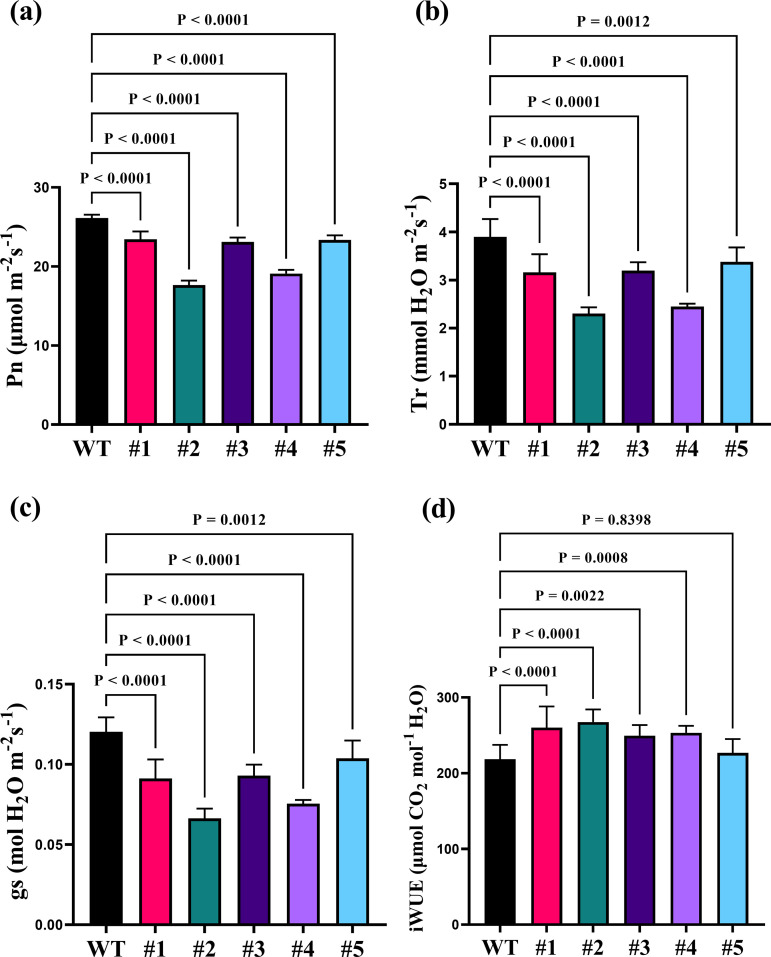
The parameters of gas exchange markedly decreased in maize *zmstomagen1/2* mutant compared with in wild type (WT). Differences of leaf net photosynthetic rate (Pn, a), transpiration rate (Tr, b), stomatal conductance (gs, c) and instantaneous water use efficiency (iWUE, d) between WT (HiII-A × HiII-B) maize and *zmstomagen1/2* mutants (#1, #2, #3, #4, #5). Data are expressed as the mean ± standard error (n ≥ 3). Results were analyzed using one-way ANOVA with Tukey’s post hoc tests at 95% confidence intervals.

The leaf net photosynthetic rate (Pn, Fig 5a), transpiration rate (Tr, Fig 5b), stomatal conductance (gs, Fig 5c) and water use efficiency (iWUE, Fig 5d) showed significant differences in maize. The Pn, Tr and gs of *zmstomagen1/2* mutant lines were significantly lower than those of WT (*P* < 0.05). However, iWUE was significantly increased in the mutant lines (Fig 5d). The photosynthetic light-response curve in *zmstomagen1/2* mutants was increased with rising light intensity. However, a reduced photosynthetic response was observed in the mutants compared to the wild type (S2 Fig). Additionally, key physiological parameters, such as the maximum net photosynthetic rate, dark respiration rate, light compensation point, and light saturation point, were all lower in the T3 mutants compared to the wild type (S2 Table).

### Differential abundance analysis of metabolic pathway profiles

**Fig 6:**
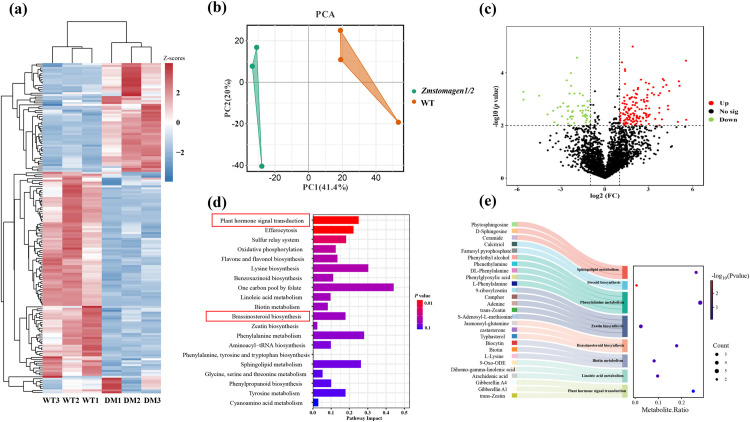
Metabolic profiling and pathway enrichment analysis in *zmstomagen1/2* mutants versus wild type. (a) Heat map showing 148 differential metabolites. The color scale represents Z-scores of metabolite abundance, with red indicating higher abundance (positive Z-scores) and blue indicating lower abundance (negative Z-scores). WT represents wild type (HiII-A × HiII-B) samples (#1, #2, and #3), while DM1, DM2, and DM3 represent *zmstomagen1/2* mutants (#1, #2, and #3), respectively. (b) Principal component analysis (PCA) plot metabolite profiles from *zmstomagen1/2* mutant and wild type (WT). Each point represents an individual sample, and the clustering patterns indicate differences in metabolite composition. (c) Volcano Plot of Differential Metabolites in *zmstomagen1/2* mutant and wild type (WT). The x-axis represents the log2 fold change in metabolite abundance between *zmstomagen1/2* mutant and WT, while the y-axis denotes the -log10 *p*-value, indicating the statistical significance of these changes. (d) Metabolic pathway enrichment analysis of differential metabolites using the KEGG database. The x-axis “Impact” represents the pathway impact value (ratio of differentially abundant metabolites to total metabolites in that pathway). The analysis shows significant enrichment in the brassinosteroid biosynthesis and zeatin biosynthesis pathways in *zmstomagen1/2* mutants. (e) Enriched metabolites between *zmstomagen1/2* mutants and WT plants, primarily associated with typhasterol, castasterone, jasmonoyl-glutamine, S-adenosyl-L-methionine, trans-zeatin, gibberellin A1, and gibberellin A4. Data are presented as mean values from three independent biological replicates, with statistical significance assessed using one-way ANOVA followed by Tukey’s post-hoc test.

To explore the effects of *zmstomagen1/2* mutants on the metabolic pathways in comparison to WT (HiII-A × HiII-B), a differential metabolic pathway analysis was constructed using liquid chromatography-mass spectrometry (LC-MS) (Fig 6). MetaboAnalyst (https://www.metaboanalyst.ca/) was utilized to generate heatmaps and identify differential metabolic pathways. 148 differential metabolites were identified across *zmstomagen1/2* mutants and WT (HiII-A × HiII-B) plant groups according to the changing levels of metabolites (Fig 6a). Principal component analysis (PCA) plot was performed to identify variation in metabolite profiles among leaf samples from *zmstomagen1/2* mutants and WT (HiII-A × HiII-B) plants (Fig 6b). Volcano plot was employed to filter metabolites of interest based on their log2(fold change) and -log10(*p* value) (Fig 6c). Additionally, an enrichment analysis of differential metabolites was performed using the KEGG database (Figs 6d, 6e and S3). There are significant changed in brassinosteroid (typhasterol, castasterone), cytokinin (trans-zeatin), and gibberellin (GA1, GA4) metabolism in the *zmstomagen1/2* mutants. Notably, the brassinosteroid biosynthesis pathway showed the highest enrichment, with typhasterol significantly upregulated in the mutants. Similarly, gibberellin A4 was also upregulated, while other metabolites such as castasterone, jasmonoyl-glutamine, S-adenosyl-L-methionine, trans-zeatin, and gibberellin A1 showed altered abundance in the mutants.

## Discussion

### STOMAGEN are conserved in mediating stomatal development

*STOMAGEN*, a positive intercellular signaling factor, regulates stomatal development in *Arabidopsis* [15]. Its sequence and phylogenetic analysis showed that there existed three homologs of *STOMAGEN* in *Zea mays L.*, named *ZmSTOMAGEN1*, *ZmSTOMAGEN2* and *ZmSTOMAGEN3*, respectively (S1 Fig). *ZmSTOMAGEN1* and *ZmSTOMAGEN2* were selected in this study for targeted gene editing because they share high similarity with the sequences of *STOMAGEN* and both of them have a signal peptide, which can guide ZmSTOMAGEN1/2 peptides to the intercellular site acting as a ligand. However, ZmSTOMAGEN3 (GRMZM2G017321/ Zm00001d049795) was deficient of the signal peptide according to the prediction of SignaIP v5.0 (http://www.cbs.dtu.dk/services/SignalP/).

The CRISPR–Cas9 system has been used extensively for genome editing due to its simplicity, efficiency, and versatility [26,27]. The double deficient mutant of *ZmSTOMAGEN1* and *ZmSTOMAGEN2* was created using CRIPR-Cas9 system in T3 maize lines. The observed reduction in *ZmSTOMAGEN1* and *ZmSTOMAGEN2* expression in mutant lines may result from multiple factors. The 5’UTR deletions in lines #2 and #4 likely disrupt critical RNA secondary structures or sequence motifs essential for transcript stability [28]. These deletions may eliminate binding sites for RNA-stabilizing proteins or alter local structural elements that protect the mRNA from degradation pathways, consistent with studies demonstrating that even small changes in 5’ UTR sequences can dramatically impact transcript accumulation in plants [29]. By confirming that the closely related *ZmEPFL4* genes were unaffected in *zmstomagen1/2* mutants (S4 Fig), we demonstrated that the observed stomatal phenotypes resulted from the loss of ZmSTOMAGEN function rather than from off-target editing of other EPF/EPFL family members.

### ZmSTOMAGEN act as positive regulators to induce stomatal formation

In contrast to other *EPF* genes, *EPFL9/STOMAGEN* acts as a positive regulator of stomatal development. It competitively binds to receptor complexes containing TMM and ER family receptors [15], inhibiting the activation of the YDA-MKK5/7-MPK3/6 pathway. This inhibition prevents the phosphorylation of the SPCH protein by MPK3/6, thereby promoting the establishment of stomatal lineage in *Arabidopsis thaliana* [3,30]. In the present study, we observed an abnormal stomata phenotype in double *zmstomagen1/2* mutant plants, characterized by reduced stomatal density and fewer stomata between epidermal cells (Fig 4). The phenotype likely results from a failure to form stomatal cell file or maintain stomatal cell fate. To investigate this, we examined the expression of transcription factors *SPCH* and *MUTE* and found a significant decreased in double *zmstomagen1/2* mutant plants (Fig 2d). This is consistent with previous research showing misoriented GMC divisions and subsidiary cell defects in plants lacking *MUTE* transcription factors [31]. Overexpressed *SPCH1/2* could induce additional divisions of epidermal cells in grass [7]. The present study revealed that *ZmSPCH1* was significantly down-regulated in the *zmstomagen1/2* maize (T3), resulting in higher proportions of smaller daughter cells or absence of stomata (Fig 4). The phenotypes observed in *zmstomagen1/2* mutants can be explained by a regulatory cascade. When *ZmSTOMAGEN1/2* was reduced, it affected the MAPK signaling pathway, which in turn down-regulated both *ZmSPCH1* and *ZmMUTE* expression. ZmSPCH1 controls the entry divisions into the stomatal lineage, while ZmMUTE regulates the differentiation of guard mother cells. Their combined down-regulation explained the abnormal stomatal phenotypes in *zmstomagen1/2* mutants. Our results suggest that additional factors are involved in regulating the distribution of stomatal cell files and rows prior to the initiation of stomatal development. Similar regulatory mechanisms have been reported in other grasses, where *bdyoda1−1* mutation affected stomatal row identity establishment in *Brachypodium distachyon* [32]. Recent research suggests that vein-derived morphogen gradients might regulate stomatal row initiation [33], though further investigation is required to confirm our findings. The observed phenotype is likely correlated with significant down-regulation of *ZmSCRM2*, which is constrained to smaller daughter cells during stomatal lineage asymmetric divisions [32]. This suggests the developmental defect likely occurred during early stages of stomatal cell file formation, prior to the generation of smaller daughter cells.

Modifying stomatal behavior in grasses represents a potential pathway to boost photosynthetic capacity of plants [2]. A previous study revealed that increased stomatal density in *STOMAGEN*‐overexpressing plants in *Arabidopsis* led to a 30% increase in photosynthetic rate [34]. However, our current study on *Zea mays L.* demonstrated that the double mutant *zmstomagen1/2* significantly suppressed stomatal density and the stomatal index, leading to an 8.6-30.3% reduction in photosynthesis (Fig 5). Plants can fine-tune carbon assimilation and enhance water use efficiency by directly targeting *EPF* genes that regulate stomatal density. For instance, overexpression the *EPF1* and *EPF2* genes in *Arabidopsis* enhanced WUE by 20% without impacting photosynthesis. Conversely, *Arabidopsis* plants lacking both *EPF1* and *EPF2* expression, as observed in *epf1epf2* mutants, exhibited higher stomatal density, higher maximum stomatal conductance, and consequently, lower WUE [35]. Furthermore, the present study indicated that *zmstomagen1/2* mutants with diminished stomatal density exhibited lower gs and Tr in maize, but enhanced instantaneous water use efficiency. The results are highly consistent with the fact that *OsEPF1* overexpression lines with fewer stomata utilized less water [13]. This robust evidence underscores the intricate relationship between stomatal density, photosynthesis, and water use efficiency, highlighting the potential for targeted genetic modifications to optimize these critical plant processes.

Stomata development in plants is involved in multiple metabolic pathways such as hormone signaling and photosynthesis [36,37]. Disruptions in stomatal development are often indicated by shifts in critical metabolites within these pathways [38]. To investigate this, we conducted a metabolic analysis of developing stomata by comparing metabolite variations between *zmstomagen1/2* mutants and wild-type maize (Figs 6 and S3). This analysis uncovered 148 metabolites that were differentially expressed between the *zmstomagen1/2* mutants and wild-type plants (Fig 6c). These metabolites were primarily enriched in 20 pathways, including plant hormone signal transduction, lysine biosynthesis, and secondary metabolite synthesis, etc. (Fig 6d).

Recent research has primarily focused on the role of brassinosteroid signaling pathways in stomatal development [39]. The *zmstomagen1/2* mutants exhibited the changes in the level of BR biosynthesis intermediates, such as typhasterol and castasterone, indicating a disruption in the BR pathway (Fig 6e). BRs regulate stomatal development through a complex signaling cascade. The pathway operates via the GSK3-like kinase BIN2 (BRASSINOSTEROID INSENSITIVE 2), which functions downstream of ERECTA family receptors and upstream of the MAPK kinase kinase (MAPKKK) YDA (also known as YODA). Under low BR conditions, BIN2 remains active and phosphorylates YODA, thereby inhibiting its function and promoting stomatal formation. When BR binds to BRI1 (BRASSINOSTEROID INSENSITIVE 1), they initiate a cascade that inactivates BIN2, enabling YDA activation and subsequent MAPK-mediated phosphorylation of SPCH, which ultimately suppresses stomatal development [40]. BIN2 can phosphorylate SPCH directly at sites overlapping with MAPK targets and at four distinct residues in the amino-terminal region [41]. This phosphorylation antagonizes SPCH activity and restricts epidermal cell proliferation. Notably, BR show context dependency, inhibiting stomatal development in leaves while enhancing it in hypocotyls [42]. The altered BR levels observed in *zmstomagen1/2* mutants likely contribute to their stomatal phenotypes through these regulatory mechanisms, although further research is required to elucidate the interaction between *ZmSTOMAGEN* and BR signaling in maize. Furthermore, *zmstomagen1/2* mutants could influence the crosstalk of zeatin and GA signaling, contributing to stomatal development abnormalities (Fig 6d and e). BRs also regulate the expression of genes involved in GA metabolism and signaling. The basic helix-loop-helix transcription factors like CES and its homologues BEE1 and BEE3 play crucial roles in GA catabolism. These transcription factors can induce the expression of GA2ox7, encoding an enzyme for GA degradation, thereby modulating GA levels in response to BR signaling [43]. These findings highlight the intricate regulatory network of brassinosteroids and other hormones in governing stomatal patterning and development.

## Conclusion

In summary, this study reveals that *STOMAGEN* acts as a positive regulator of stomatal number in maize by suppressing the expression of *SPCH1*, *MUTE* and *SCRM2*. This finding advances our understanding of stomatal development regulation. The knockdown of *ZmSTOMAGEN1/2* leads to a reduction in stomatal density on the leaf epidermis, thereby improving plant WUE. Comprehensive metabolic analysis of *zmstomagen1/2* mutants identified significant alterations in 148 metabolites compared to wild type, particularly those in the brassinosteroid and zeatin biosynthesis pathways. These modifications suggest a disruption in the brassinosteroid signaling pathway, providing a valuable approach to manipulate stomatal density in transgenic plants through precise control of *ZmSTOMAGEN1/2* expression levels.

## Supporting information

S1 Fig Evolutionary tree analysis of ZmSTOMAGEN. (TIF)

S2 Fig Photosynthetic light-response curves (Pn-PAR) of maize leaves in WT and mutants (T1). (TIFF)

S3 Fig Differential metabolite abundance of key metabolites in *zmstomagen1/2* mutants compared to wild type (WT). (TIF)

S4 Fig Relative expression levels of *ZmEPFL4−1*, *ZmEPFL4−2*, and *ZmSTOMAGEN3* genes in wild-type (HiII-A × HiII-B) and *zmstomagen1/2* mutants. (TIF)

S1 Table Primers used for gene-specific RT-qPCR and hybridization. (DOCX)

S2 Table Comparison of the photosynthetic-parameters fitted by non-rectangular hyperbola models in maize wild type and *zmstomagen1/2* mutants. (DOCX)

S1 Data Raw data for all figures. (XLSX)

S1 Raw Images Original uncropped and unadjusted gel images supporting Fig 2b. (PDF)
