# Supplementary figures and images for: The regulatory role of ZmSTOMAGEN1/2 in maize stomatal development is elucidated via gene editing and metabolic profiling
Source: PLoS One. 2025 Jul 14;20(7):e0328433. doi: 10.1371/journal.pone.0328433 (PMC12258594; doi:10.1371/journal.pone.0328433)

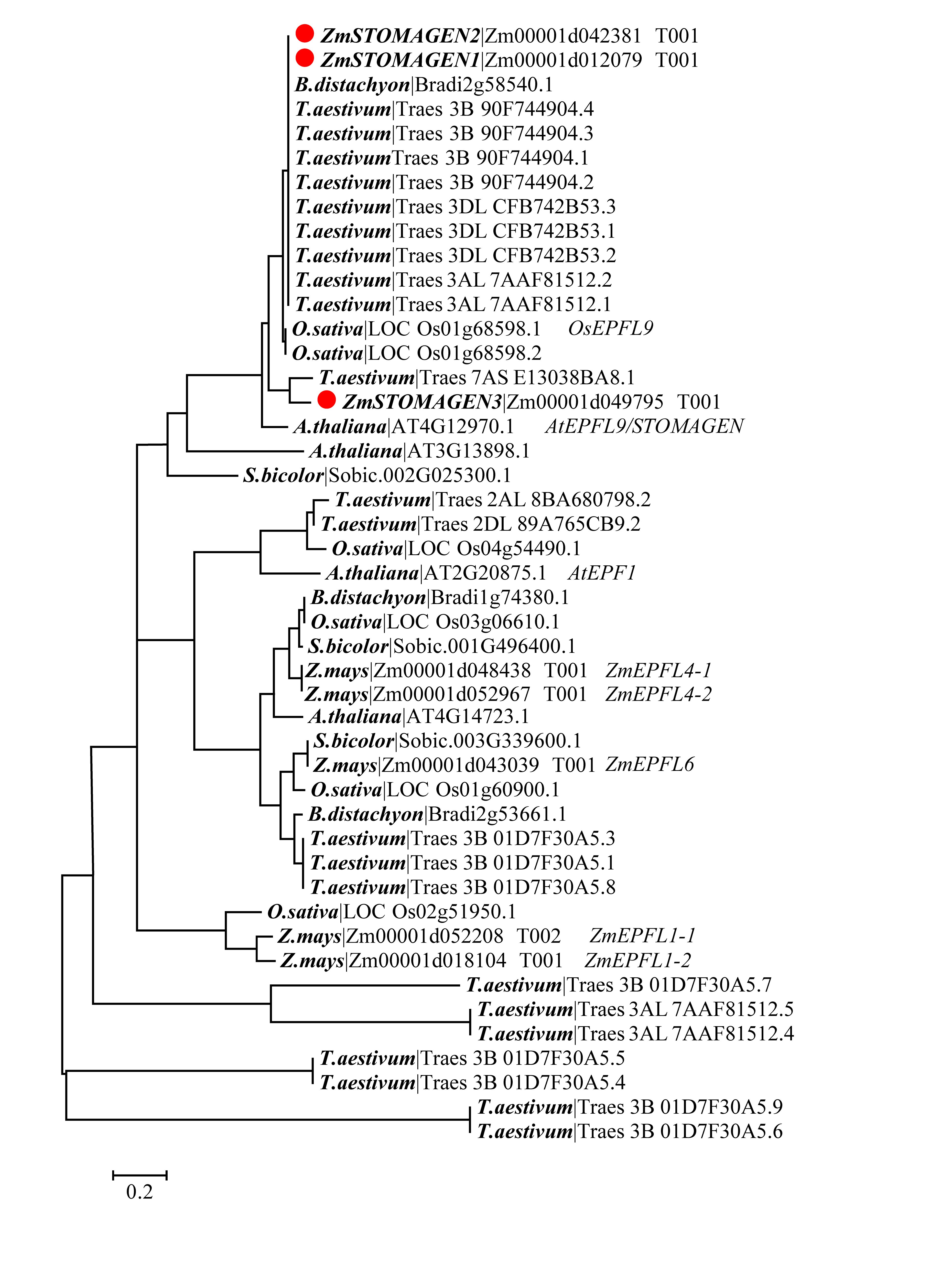

Supplement: S1 Fig — (TIF) [file pone.0328433.s001.tif]

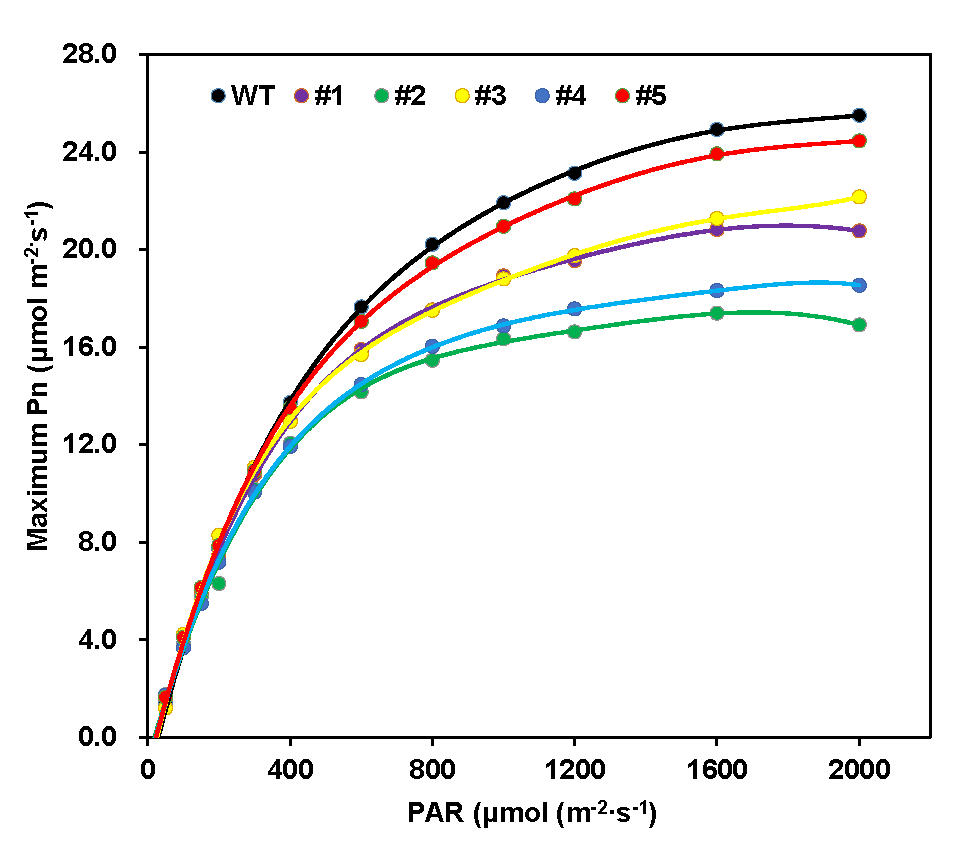

Supplement: S2 Fig — (TIFF) [file pone.0328433.s002.tiff]

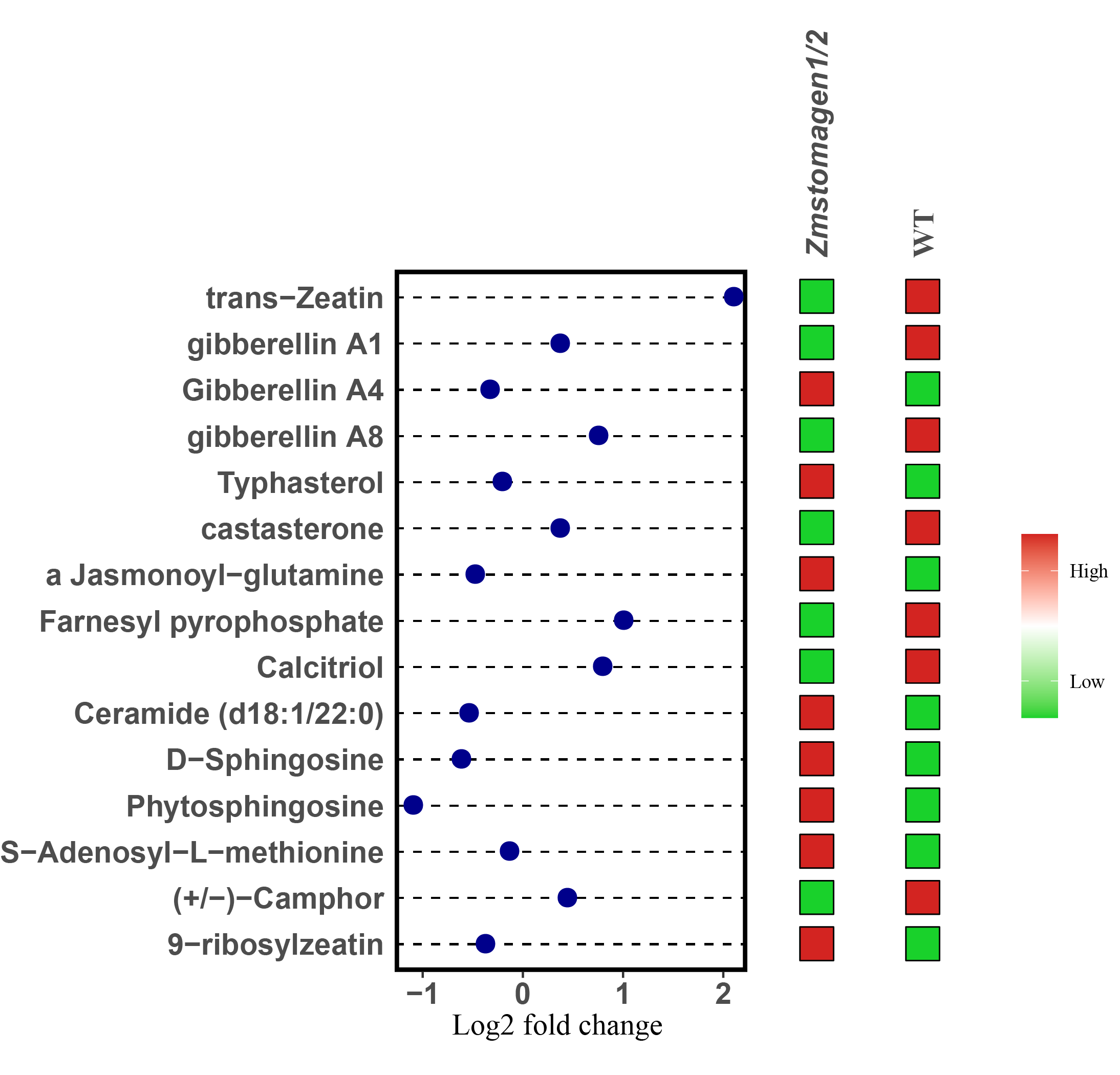

Supplement: S3 Fig — (TIF) [file pone.0328433.s003.tif]

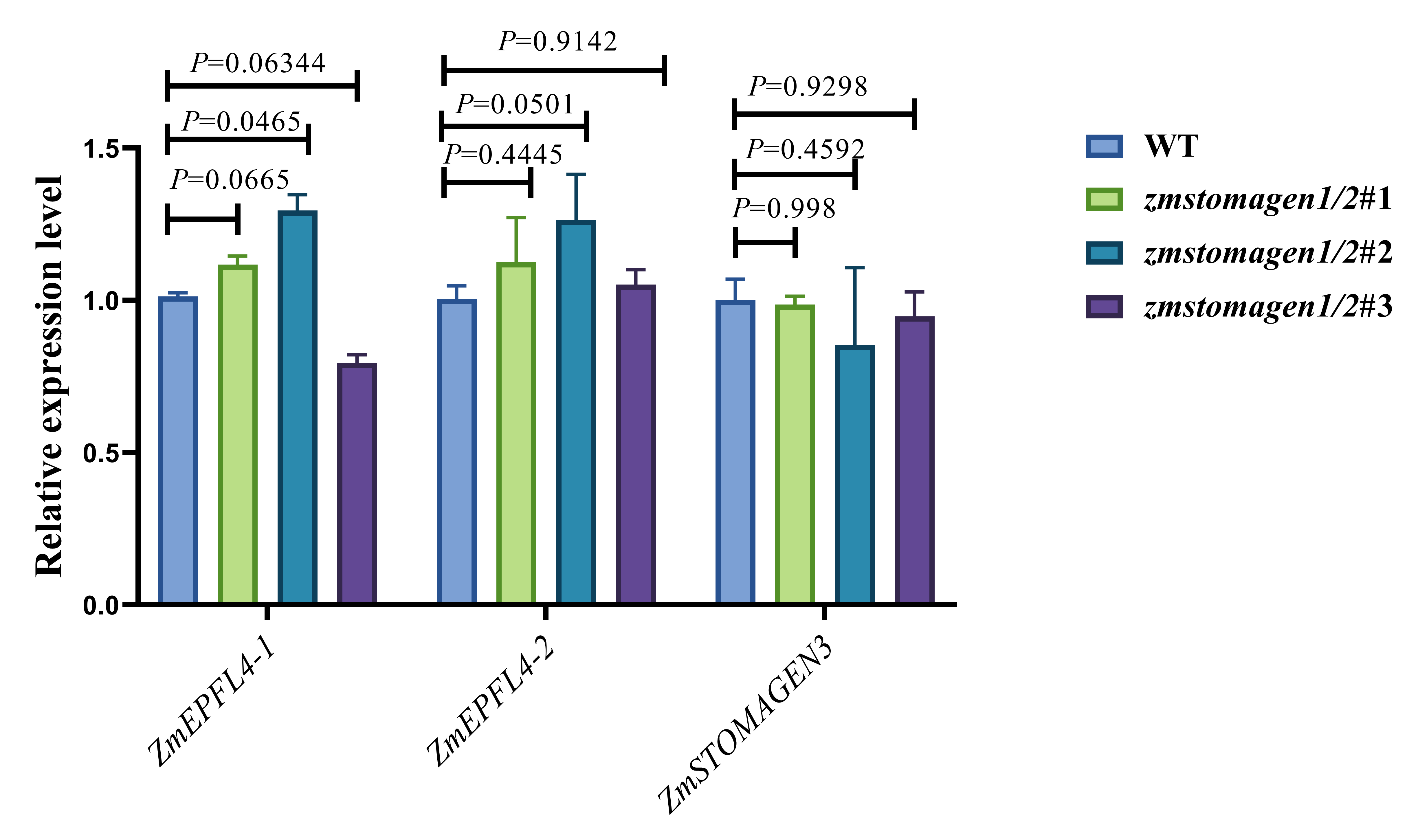

Supplement: S4 Fig — (TIF) [file pone.0328433.s004.tif]
