# Supplementary material for: The regulatory role of ZmSTOMAGEN1/2 in maize stomatal development is elucidated via gene editing and metabolic profiling
Source: PLoS One. 2025 Jul 14;20(7):e0328433. doi: 10.1371/journal.pone.0328433 (PMC12258594; doi:10.1371/journal.pone.0328433)
Supplement: S1 Table — (DOCX) [file pone.0328433.s005.docx]

**S1Table.** Primers used for gene-specific RT-qPCR and hybridization

| Primers | Sequence (5’ to 3’) |
| --- | --- |
| Actin-F | GGGATTGCCGATCGTATGAG |
| Actin-R | GAGCCACCGATCCAGACACT |
| ZmSTOM1-F | ATGGCTAATGGTTGCCCCACAT |
| ZmSTOM1-R | TTAGACCCTTGATGACCTTCGG |
| ZmSTOM2-F | ATGGCTAATGGTTGCTCCACGA |
| ZmSTOM2-R | GCCTTTGTTTAGACCCTTGACG |
| SCRM2-F | CCCATCAACACCGACATTGC |
| SCRM2-R | CCTGACTTCGACTGTGGCTT |
| SPCH1-F | GGGCAGACTGTCAAGGTCAT |
| SPCH1-R | CGCTGAGCTCGCACTTTATT |
| MUTE-F | AGGTGCTCCACCTCAACATC |
| MUTE-R | CCTCGTAGGCAAGATCCTCA |
| SDD1-F | GTCCAACTTCACCGTGCTCT |
| SDD1-R | CGGTCGATTATGTCAGCCGT |
| EPFL4-1-F | GGACGGCTACTCCTGGTTCT |
| EPFL4-1-R | GACAGTGAGTGAGCGAAGCA |
| EPFL4-2-F | TGGATTGATCGAGACGACGG |
| EPFL4-2-R | TACGCACGCCTAATCACCTC |
| In situ hybridization |  |
| ZmSTOM1-T7-F | TAATACGACTCACTATAGGAATGGCTAATGGTTGCCCCACAT |
| ZmSTOM1-T3-R | AATTAACCCTCACTAAAGGGTTAGACCCTTGATGACCTTCGG |
| ZmSTOM2-T7-F | TAATACGACTCACTATAGGAATGGCTAATGGTTGCTCCACGA |
| ZmSTOM2-T3-R | AATTAACCCTCACTAAAGGGGCCTTTGTTTAGACCCTTGACG |
