# Supplementary material for: The regulatory role of ZmSTOMAGEN1/2 in maize stomatal development is elucidated via gene editing and metabolic profiling
Source: PLoS One. 2025 Jul 14;20(7):e0328433. doi: 10.1371/journal.pone.0328433 (PMC12258594; doi:10.1371/journal.pone.0328433)
Supplement: S2 Table — (DOCX) [file pone.0328433.s006.docx]

**S2 Table.** Comparison of the photosynthetic-parameters fitted by non-rectangular hyperbola models in maize wild type and *zmstomagen1/2* mutants. Each value is the mean of 15 replications and the determination coefficient (*R*^2^) is listed for each model.

| Photosynthetic parameters | Wild type | *zmstomagen1/2* mutants | | | | |
| --- | --- | --- | --- | --- | --- | --- |
|  |  | 2-1 | 2-2 | 2-3 | 2-4 | 2-5 |
| Maximum net photosynthetic rate (μmol m^-2^·s^-1^) | 26.769 | 21.857 | 18.250 | 22.705 | 19.459 | 25.668 |
| Dark respiration rate (μmol m^-2^·s^-1^) | 1.970 | 1.447 | 1.174 | 1.562 | 1.381 | 1.622 |
| Light compensation point (μmol m^-2^·s^-1^) | 36 | 28 | 24 | 28 | 28 | 32 |
| Light saturation point (μmol m^-2^·s^-1^) | 1512 | 1268 | 1088 | 1316 | 1160 | 1488 |
| Apparent quantum yield (molmol^-1^) | 0.055 | 0.053 | 0.051 | 0.053 | 0.051 | 0.053 |
| Correlation coefficient *R^2^* | 1 | 0.999 | 0.997 | 0.999 | 1 | 1 |
