# Supplementary material for: The regulatory role of ZmSTOMAGEN1/2 in maize stomatal development is elucidated via gene editing and metabolic profiling
Source: PLoS One. 2025 Jul 14;20(7):e0328433. doi: 10.1371/journal.pone.0328433 (PMC12258594; doi:10.1371/journal.pone.0328433)

Method: PCR amplification

Gel: 1% agarose gel

Figure Panel: Figure 2 Panel b upper
Loading order: L1-L9 (left to right)

Lane Information:

L1 (M): DNA Marker

L2 (+): Empty vector control

L3 (WT): Wild type

L4 (#1): ZmSTOMAGEN1/2 knockout mutant line #1

L5 (#2): ZmSTOMAGEN1/2 knockout mutant line #2

L6 (#3): ZmSTOMAGEN1/2 knockout mutant line #3

L7 (#4): ZmSTOMAGEN1/2 knockout mutant line #4

L8 (#5): ZmSTOMAGEN1/2 knockout mutant line #5

L9 (#6): ZmSTOMAGEN1/2 knockout mutant line #6

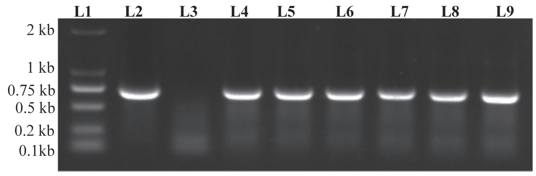

Method: PCR amplification

Gel: 1% agarose gel

Figure Panel: Figure 2 Panel b middle

Loading order: L1-L9 (left to right)

Lane Information:

L1 (M): DNA Marker

L2 (WT): Wild type

L3 (-): Water instead of maize DNA

L4 (#1): ZmSTOMAGEN1/2 knockout mutant line #1

L5 (#2): ZmSTOMAGEN1/2 knockout mutant line #2

L6 (#3): ZmSTOMAGEN1/2 knockout mutant line #3

L7 (#4): ZmSTOMAGEN1/2 knockout mutant line #4

L8 (#5): ZmSTOMAGEN1/2 knockout mutant line #5

L9 (#6): ZmSTOMAGEN1/2 knockout mutant line #6

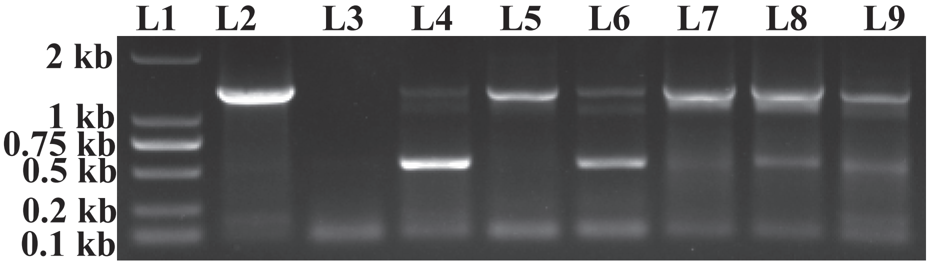

Method: PCR amplification

Gel: 1% agarose gel

Figure Panel: Figure 2 Panel b lower

Loading order: L1-L9 (left to right)

Lane Information:

L1 (M): DNA Marker

L2 (WT): Wild type

L3 (-): Water instead of maize DNA

L4 (#1): ZmSTOMAGEN1/2 knockout mutant line #1

L5 (#2): ZmSTOMAGEN1/2 knockout mutant line #2

L6 (#3): ZmSTOMAGEN1/2 knockout mutant line #3

L7 (#4): ZmSTOMAGEN1/2 knockout mutant line #4

L8 (#5): ZmSTOMAGEN1/2 knockout mutant line #5

L9 (#6): ZmSTOMAGEN1/2 knockout mutant line #6

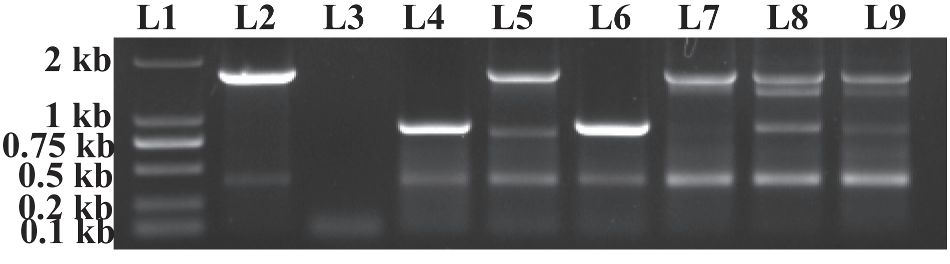

Supplement: S1 Raw Images — (PDF) [file pone.0328433.s008.pdf]
